# Temporal and Motor Representation of Rhythm in Fronto-Parietal Cortical Areas: An fMRI Study

**DOI:** 10.1371/journal.pone.0130120

**Published:** 2015-06-15

**Authors:** Naho Konoike, Yuka Kotozaki, Hyeonjeong Jeong, Atsuko Miyazaki, Kohei Sakaki, Takamitsu Shinada, Motoaki Sugiura, Ryuta Kawashima, Katsuki Nakamura

**Affiliations:** 1 Primate Research Institute, Kyoto University, Kanrin 41–2, Inuyama-city, Aichi, 484–8506, Japan; 2 Institute of Development, Aging and Cancer (IDAC), Tohoku University, Seiryo-machi 4–1, Aoba-ku, Sendai, 980–8575, Japan; University of Maryland, College Park, UNITED STATES

## Abstract

When sounds occur with temporally structured patterns, we can feel a rhythm. To memorize a rhythm, perception of its temporal patterns and organization of them into a hierarchically structured sequence are necessary. On the other hand, rhythm perception can often cause unintentional body movements. Thus, we hypothesized that rhythm information can be manifested in two different ways; temporal and motor representations. The motor representation depends on effectors, such as the finger or foot, whereas the temporal representation is effector-independent. We tested our hypothesis with a working memory paradigm to elucidate neuronal correlates of temporal or motor representation of rhythm and to reveal the neural networks associated with these representations. We measured brain activity by fMRI while participants memorized rhythms and reproduced them by tapping with the right finger, left finger, or foot, or by articulation. The right inferior frontal gyrus and the inferior parietal lobule exhibited significant effector-independent activations during encoding and retrieval of rhythm information, whereas the left inferior parietal lobule and supplementary motor area (SMA) showed effector-dependent activations during retrieval. These results suggest that temporal sequences of rhythm are probably represented in the right fronto-parietal network, whereas motor sequences of rhythm can be represented in the SMA-parietal network.

## Introduction

People feel a rhythm when the sounds reach their ears with a systematic pattern. There are two different notions regarding neural representation of rhythm. To memorize a certain rhythm, it is necessary to perceive its temporal patterns and organize them into a hierarchically structured sequence, according to certain principles [1]. Some brain imaging studies have reported that cortical regions, such as the superior temporal cortex and the inferior frontal cortex, exhibit activations related to the metrical or non-metrical structures during rhythm perception [2,3] or retention [4]. These brain activations are thought to be related to regular perceptual accent [2], suggesting the possibility of temporal representation of rhythm.

On the other hand, rhythm perception often provokes an involuntary or unintentional movement. Indeed, some previous studies have suggested that perception of rhythmic stimuli automatically recruits brain regions, such as the premotor cortex, supplementary motor area (SMA), and cerebellum [2,3,5], that play an important role in motor control. These data suggest that rhythm information is represented and retained as information regarding sequences of bodily movements in the motor system.

Based on these previous studies, we hypothesized that the rhythm information is represented in two ways: temporal and motor sequences. The motor sequences depend on effectors whereas the temporal sequences are effector-independent. We examined the degree of dependence on the effectors for activation in each brain region during rhythm processing to distinguish the brain regions responsible for the representation of the temporal and motor sequences.

There are a few studies that investigated the effector-independent activations related to rhythm tapping [6,7,8]. The study by Meister et al. reported brain activations during tapping or articulation at a constant rate, and that by Bengtsson et al. described activations during synchronization and reproduction of well-trained rhythmic sequences. They observed overlapping activations in the dorsal premotor cortex, superior temporal gyrus, SMA, and cerebellum for both speech and finger tapping. However, both of these studies mainly investigated the reproduction processes rather than the encoding processes.

We previously reported that the inferior frontal cortex, inferior parietal cortex and SMA are implicated in the encoding and retrieval of rhythm information using a working memory task [9]. However, it is unclear whether these brain regions represent the temporal or motor sequences of rhythm information. It is also unknown how the rhythm information is encoded and used to reproduce the memorized rhythm. Here, we used the same working memory task as previously used, but in the present study, we requested from participants to use four different effectors to dissociate the neural substrates involved in temporal and motor representations, and to reveal the neural networks of these representations.

## Materials and Methods

### 2.1 Participants

Twenty-nine healthy Japanese volunteers participated in this experiment (15 females and 14 males; average age: 21.4 years; range: 18–25 years). All participants were right-handed with no neurological or psychiatric history. They were recruited from the Tohoku University community, and written informed consent was obtained from each participant. This study was approved by the Human Research Ethics Committee of the Primate Research Institute and Institutional Review Board of the Tohoku University School of Medicine. Data from six participants were excluded from any additional analyses, because of head motion (three participants), technical failure of data acquisition (one participant), and poor performance in a specific session (two participants).

### 2.2 Behavioral tasks

**Fig 1:**
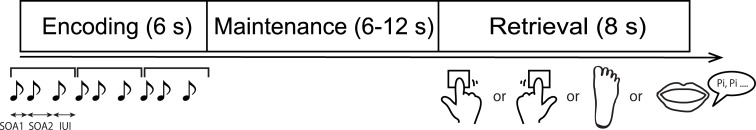
Rhythm working memory tasks. A sample rhythm pattern was presented at the beginning of each trial. Each rhythm pattern consisted of three repeats of a three-pure-tone sequence. The participants were required to memorize the rhythm pattern within 6 s (encoding phase), to maintain the rhythm information for 6–12 s (maintenance phase), and reproduce it by tapping with the right index finger, left index finger or right foot, or by articulation within 8 s (retrieval phase). We used 20 rhythmic patterns for each participant. The two out of three durations (SOA1, SOA2 and IUI) in each pattern were constantly same. The SOA and IUI were chosen from six possible durations (0.4, 0.5, 0.6, 0.8, 1.0 or 1.2 s) so that the duration of each rhythmic pattern ranged from 4 to 6 s. Abbreviations: SOA, stimulus onset asynchrony; IUI, inter-unit-interval.

We conducted a rhythm working memory (RHY) task, where the participant had to reproduce a rhythmic pattern using any of four effectors; the right index finger, the left index finger, the right foot, or the mouth (Fig 1). We also performed a number working memory (NUM) task as a control. The experimental session comprised four runs, and each run consisted of four blocks corresponding to the four effectors. The participants used the same effector throughout a block of 10 trials. Before the first trial for each block, an instructional cue was presented and the participants could recognize which effector to use.

At the beginning of each trial of the RHY task, a white fixation cross was presented at the center of a computer monitor. After 1 second, a rhythmic pattern was presented. The participant was required to memorize the rhythmic pattern within 6 s (defined as the encoding phase: E phase), maintain it for 6–12 s (maintenance phase: M phase), and then reproduce it by one of the four different effectors; (1) tapping with the right index finger, (2) tapping with the left index finger, (3) tapping with the right foot toe, or (4) making the 'pi' sound by the mouth within 8 s (retrieval phase: R phase).

Each rhythmic pattern comprised of nine 2000-Hz pure tones with a duration of 0.1 s; three repeats of a three-tone unit, which induced a perceptual accent at the beginning of each unit. We defined a stimulus onset asynchrony (SOA) as a duration from the onset of a tone to the onset of the next tone, and defined an inter-unit-interval (IUI) as a duration from the onset of the 3^rd^ tone in the prior unit to the onset of the 1^st^ tone in the next unit. Each three-tone unit contains the two SOAs (See Fig 1). We used 20 rhythmic patterns for each participant. The two out of three durations (two SOAs and IUI) in each pattern were constantly same (0.5–0.5–1.0–0.5–0.5–1.0…, 1.2–0.4–0.4–1.2–0.4–0.4…, etc.). The SOA and IUI were chosen from six possible durations (0.4, 0.5, 0.6, 0.8, 1.0 or 1.2 s) so that the duration of each rhythmic pattern ranged from 4 to 6 s. The participants listened to the rhythmic patterns through MRI-compatible headphones.

At the beginning of each trial of the NUM task, a red fixation cross was presented. The participants could easily recognize the type of task (RHY or NUM) by the color of the fixation cross, which was presented continuously throughout the trial. After 1 second, a tone sequence was presented within 6 s of the E phase and participants were required to memorize the number of tones in the sequence. After 6–12 s of the M phase, the participants were expected to replicate the number of tones with the effector, which was instructed beforehand, regardless of the tempo within 8 s of the R phase. For one participant only, the duration of the R phase of both RHY and NUM tasks was set to 7 s. In the NUM task, the tone sequence composed of seven to eleven 2000-Hz pure tones with a duration of 0.1 s. The SOAs were constant at 0.5 s. The duration of each sequence ranged from 3.1 to 5.1 s. We could control the effects of sensory stimulation, working memory load, and tapping actions on the brain activity with the NUM tasks.

The RHY and NUM tasks were intermingled pseudo-randomly. In all trials, a Kanji character representing "next" was presented at the center of the monitor so that the participants could recognize the end of the ongoing trial. The 2-s interval from the onset of “next” to the onset of the fixation cross was an inter-trial interval. During the inter-trial interval, participant could relax, but were instructed to keep their eyes focused on the center of the monitor.

For both tasks, if the number of taps or articulation was different from the number of tones in a trial, we regarded the trial as erroneous. Prior to scanning, all participants performed a practice test block by using their right finger to ensure that they all understood the procedure for both RHY and NUM tasks. One block consisted of 10 trials. All participants met a criterion (more than eight correct trials in a block) for the first test block. Behavioral tasks and data collection were controlled by a script written in MATLAB (MathWorks, Natick, MA, USA) using the Psychophysics Toolbox extensions (http://psychtoolbox.org/).

### 2.3 Behavioral data analysis

We calculated the percentages of erroneous trials for each block of the RHY and NUM tasks and compared the degree of difficulty among effectors using a Kruskal-Wallis test. In addition, to evaluate the accuracy of reproduction in the RHY task, we calculated a “get-into-rhythm” (GIR) index [9], modified from [10] for each participant by using the data from each trial according to the following equation;

$$\text{GIR index }=\;\overset{8}{\underset{\text{i}}{\sum }}\frac{\left|\text{ROA}—\text{SOA}\right|}{\text{SOA}}/8$$

where ROA is the response (tapping or articulation) onset asynchrony, (i.e., the time from the onset of a response to the onset of the next response), and SOA is the stimulus onset asynchrony, (i.e., the time from the onset of a stimulus (tone) to the onset of the next stimulus). The response data for finger and foot tapping were obtained from MRI-compatible buttons. The audio data were recorded through a noise-canceling microphone and analyzed by a script written in MATLAB. The GIR index indicated the mean of the normalized jitters or inaccuracies during the eight intervals in each trial. The GIR index value would be close to zero if the performance of a participant was optimal, whereas the index value would be large when the performance was poor. We compared the GIR index values of the RHY tasks among the different effectors using a one-way ANOVA.

### 2.4 Image acquisition

For fMRI measurements, gradient echo T2*-weighted echo-planar images with blood oxygenation level-dependent (BOLD) contrast were obtained using 3.0 Tesla MRI scanner (Achieva Quasar Dual, Philips, The Netherlands) with the participants in a supine position. The participant was instructed not to move their head and body, except for the right index finger, left index finger, right foot, or mouth throughout the experiment.

Whole-brain volumes were collected in 40 axial slices (repetition time (TR) = 2500 ms; echo time (TE) = 25 ms; flip angle (FA) = 81°, field of view (FOV) = 192 mm, matrix size = 64 × 64; slice thickness = 3 mm; interslice gap = 1 mm; voxel size = 3 × 3 × 4 mm). The initial 3 scans of each participant were dummy scans to equilibrate the state of magnetization and were discarded from the time-series data. Thus, we collected 455 or 475 scans during the fMRI measurement. In addition, high resolution T1-weighed structural MR images (TR = shortest; TE = shortest; FOV = 240 mm; matrix size = 240 × 240; 162 sagittal slices of 1-mm thickness) were also acquired.

### 2.5 fMRI data analysis

Processing and statistical analyses of the fMRI data were performed using SPM8 (http://www.fil.ion.ucl.ac.uk/). All scans were realigned using the first scan of the experimental session as a reference to correct the effect of head motion. All functional images for each participant were slice-time corrected to a slice captured halfway through image acquisition, in order to correct for temporal differences between slices acquired early and those acquired later, in the image series. The number of the slices were 40 and besides an ascending order, slices were scanned interleaved. Then, the data were spatially normalized to the Montreal Neurological Institute (MNI) template. Each scan was spatially smoothed with a Gaussian filter (8-mm full width at half maximum) to reduce noise and minimize the effect of normalization errors.

The time-series data from each participant were applied to a voxel-by-voxel multiple regression analysis. We defined 16 conditions of interest; three-way factorial design with TASK (RHY or NUM), EFFECTOR (right finger, left finger, right foot or mouth), and PHASE (E or R phase) as factors. In our previous study, we found no significant brain activation related to rhythm processing during the M phase (Konoike et al., 2012). In the present study, the M phase was omitted from the model. These 16 conditions were separately modeled, in order to distinguish brain activations associated with each processing and each effector. The onset time and the duration of each phase (6 s for the E phase and 8 s for the R phase) were also included in this model. In addition, six-dimension head motion parameters were included. All of these models were convolved with the hemodynamic response function (HRF).

A second-level random effect analysis, using a one-sample *t*-test, was applied to appropriate parameter estimates or their contrasts.

First, to identify the brain regions related to processing of rhythm information, we used contrasts: (RXr—NXr), (RXl—NXl), (RXf—NXf), or (RXm—NXm) in each phase, where 'X' describes the E or R phase; 'R', RHY task; 'N', NUM task; 'r', right finger; 'l', left finger; 'f', foot; and 'm', mouth. Thus, REr represents RHY-Encoding-right finger. All voxel values were thresholded at *P* < 0.001 (uncorrected) and then corrected for control of the family-wise error (FWE) to *P* < 0.05 using the cluster size assuming the whole brain as a search area. To exclude brain activations due to deactivations in the NUM task, we examined significant activations within the voxels that exhibit activation compared to the baseline activity (*P* < 0.05, uncorrected) in the RHY task (inclusive mask).

Second, to identify the common activated brain regions for the four effectors, we conducted a conjunction analysis using the contrasts (RXr—NXr), (RXl—NXl), (RXf—NXf), and (RXm—NXm) for each phase. The four contrasts were separately thresholded at *P* < 0.001 uncorrected, then conjoined, and a cluster analysis was performed on these data (*P* < 0.05, corrected). We obtained another set of images of these contrasts: RXr, RXl, RXf, and RXm with statistical threshold *P* < 0.05 (uncorrected), and used the set of images as an inclusive mask in the conjunction analysis to exclude brain activations due to deactivations in the NUM task.

We found that the activation pattern under the mouth condition differed from the pattern under the other effector conditions. Therefore, we conducted an additional conjunction analysis using contrasts (RXr—NXr), (RXl—NXl), and (RXf—NXf) to identify the common brain regions activated under the right finger, left finger, and right foot conditions (*P* < 0.05, corrected). We used the mask (RXr, RXl and RXf, *P* < 0.05, uncorrected) in the additional conjunction analysis to exclude brain activations due to deactivations in the NUM task.

Finally, we want to identify brain regions associated with effector-specific processing of rhythm information, we compared the rhythm-related activity for each effector with those for the other effectors. For the search of the right index- specific region, for example, we used a contrast (RXr—NXr)–[(RXl—NXl) + (RXf—NXf) + (RXm—NXm)] for each phase (these contrast weights were set [3 –1 –1 –1], *P* < 0.05, corrected). The effector-specific activations were examined within the voxels exhibiting activations in RXr (inclusive mask, *P* < 0.05, uncorrected).

To investigate rhythm processing-related connectivity modulated by task conditions between a seed region (one of the effector-dependent or independent regions) and other brain regions, we conducted a psychophysiological interaction (PPI) analysis using SPM8 (Friston et al., 1997). We extracted volume of interest (VOI) data for the effector-independent and effector-dependent regions from the conjunction analysis (see Table 1), and defined these areas as the seed regions with a radius of 6 mm. For the regression analysis, three regressors were included; (1) a main effect of the deconvolved BOLD time series from the seed region as regional response, (2) a main effect of the psychological variable of interest as experimental input, and (3) the interaction term between the first and second regressors. In addition, six-dimension head motion parameters were also included. These regressors were convolved with the canonical HRF, and then entered into a separate regression model for each condition. The PPI analysis was conducted for each participant, and the resulting contrast images were entered in a group analysis. The statistical threshold was set at voxel-wise *P* < 0.01 uncorrected with an extent threshold of cluster-wise *P* < 0.05 corrected. For the PPI analysis of the encoding phase, psychological variables were specified by the contrasts of RHY-NUM for all three effectors; right finger, left finger and right foot conditions. We found no significant connectivity for analysis of the retrieval phase when the effectors were combined, therefore the psychological variables were separately specified for each effector of the retrieval phase.

**Table 1:** Effector-independent and dependent regions revealed by conjunction analyses.

phase	Brain region	size	*p*	*T*	xyz (MNI)
Conjunction: (RXr—NXr) ∩ (RXl—NXl) ∩ (RXf—NXf) ∩ (RXm—NXm)					
Encoding	Rt. inferior parietal lobule	249	0.048	4.73	52–36 48
	Rt. inferior frontal gyrus	187	0.040*	4.70	52 14 22
Retrieval	Rt. inferior frontal gyrus	281	0.033	5.98	52 12 22
	Rt. inferior parietal lobule	309	0.024	4.38	52–36 48
Conjunction: (RXr—NXr) ∩ (RXl—NXl) ∩ (RXf—NXf)					
Encoding	Rt. inferior frontal gyrus	206	< 0.001	5.91	50 14 24
	Lt. inferior frontal gyrus	429	0.007	5.37	-50 8 22
	Rt. inferior parietal lobule	411	0.008	5.16	50–38 48
Retrieval	Rt. inferior frontal gyrus	343	0.015	5.93	52 12 22
	Rt. inferior parietal lobule	925	< 0.001	5.54	50–46 50
	Rt. supplementary motor area	607	0.001	4.95	2 14 56
	Lt. inferior parietal lobule	345	0.016	4.27	-48–44 46

P values are cluster-wise significance.

* The cluster was observed at the threshold of *P* < 0.05 (corrected) using the peak level (not the cluster size) based on the *a priori* hypothesis that the right inferior frontal gyrus was involved in rhythm processing (Konoike et al., 2012).

## Results

### 3.1 Behavioral data

**Fig 2:**
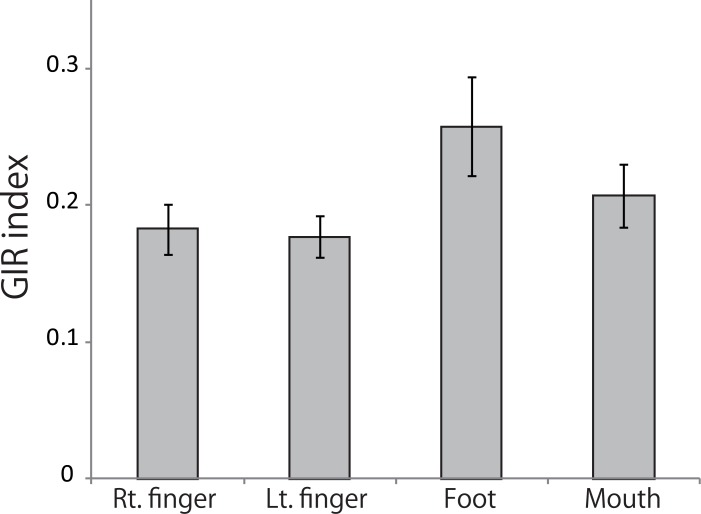
Graph demonstrating the accuracy of the rhythm reproduction. The graph shows the mean GIR index value in each effector condition of rhythm working memory tasks. A one-way ANOVA revealed that the accuracy of rhythm reproduction was consistent among these 4 effectors. Error bars indicate the standard error of the mean. See Methods for details of the GIR index. Abbreviations: Rt., right; Lt., left.

To evaluate differences in task difficulty among all four effectors, we calculated and compared the percentage of erroneous trials for each condition. In the RHY tasks, the mean percentage of erroneous trials and standard deviations for the right finger, left finger, right foot, and mouth conditions were 5.4 ± 6.7, 6.1 ± 6.0, 12.6 ± 7.5, and 5.2 ± 6.8, respectively. A Kruskal-Wallis test revealed that the percentage of erroneous trials was significantly different among the four effector conditions (H = 15.69, *P* = 0.001). A post-hoc analysis showed that the performance in the right foot condition was worse than that of either the right finger or mouth condition (*P* = 0.003 and *P* = 0.003, respectively, rank sum difference test for multiple comparison). In the NUM task, the mean percentage of erroneous trials and standard deviations for the right finger, left finger, right foot, and mouth conditions were 6.7 ± 7.9, 5.4 ± 5.2, 13.3 ± 10.9, and 3.0 ± 3.3. In the NUM task, there was also a significant difference in the error percentage among the effector conditions (H = 13.12, *P* = 0.004) and the performance in the foot condition was worse than that of the mouth condition (*P* = 0.002). Almost all the erroneous trials in the right foot condition of both RHY and NUM trials were concentrated in the first and second sessions. In addition, for a few participants, the performance was worse in the initial sessions. Indeed, the percentage of erroneous trials in the latter half of the sessions was not different among the four effector conditions (*P* = 0.258). These results suggest that for some participants, foot tapping required much practice probably because a cutaneous sensation of a foot is less than fingers. The accuracy of rhythm reproduction in the RHY tasks was assessed based on the GIR index values. The mean GIR index value and standard errors for the right finger, left finger, right foot, and mouth conditions were 0.18 ± 0.02, 0.18 ± 0.02, 0.26 ± 0.04, and 0.21 ± 0.02, respectively (Fig 2). A one-way ANOVA revealed no significant differences between effectors (*P* = 0.085). In addition, we calculated the coefficient of variation (CV) of the GIR index for each effector and examined the relationship between the GIR index and CV. We found no statistically significant correlation between the GIR index and CV in all effectors (*P* > 0.05, Spearman rank correlation coefficient).

The performance in the foot condition was worse than that in other conditions, especially in the beginning of the experiment. However, the analysis of accuracy of rhythm production suggests that the performance was not significantly different among the four effector conditions.

### 3.2 fMRI data

#### Effector-independent/dependent activations during rhythm encoding and retrieval

**Fig 3:**
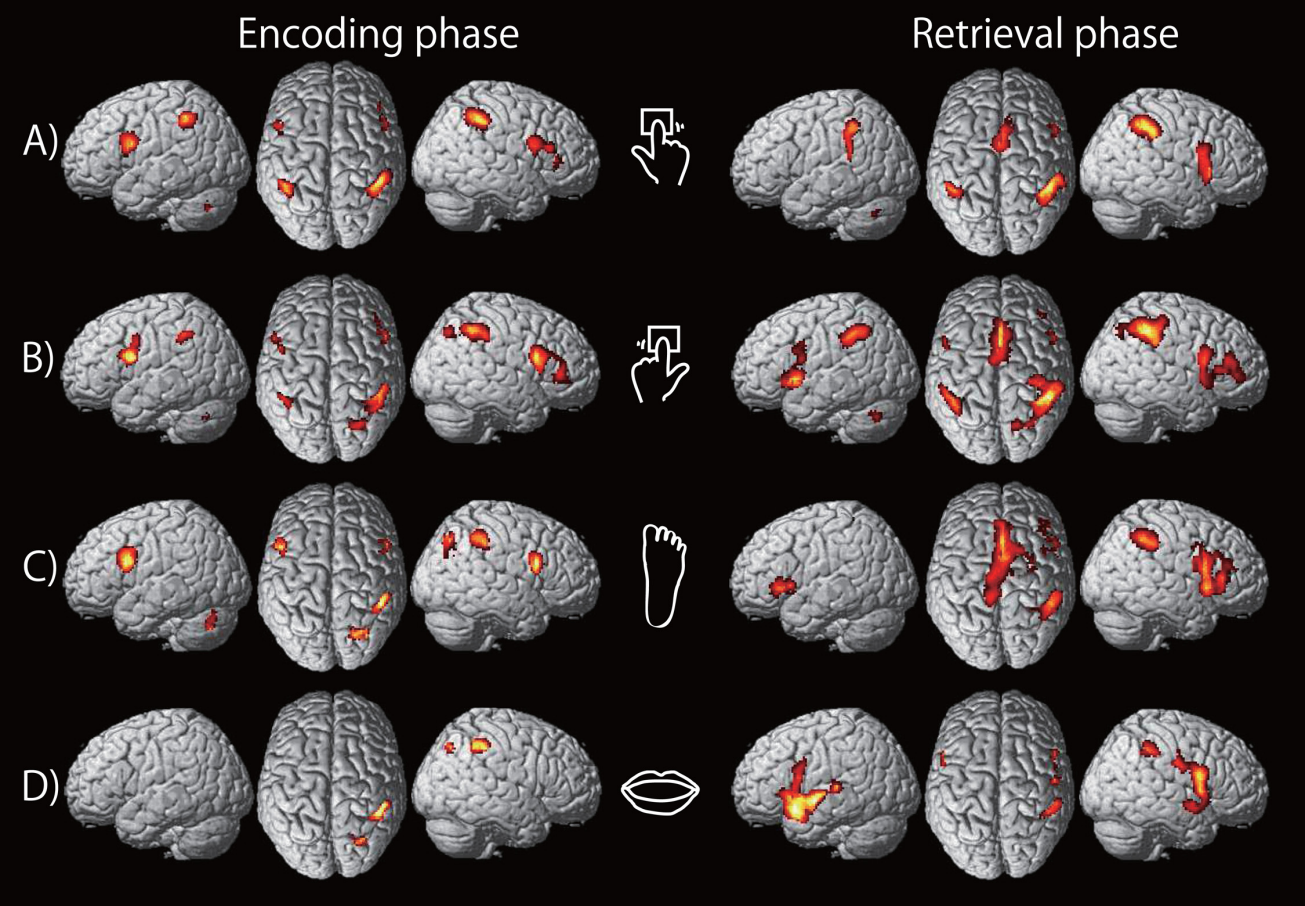
Cortical brain regions exhibiting significant activations during the encoding (left panel) and the retrieval (right panel) phases of the rhythm working memory tasks compared to the number working memory tasks (threshold: *P* < 0.05; FWE-corrected of the clusters). A) right finger, B) left finger, C) foot, and D) mouth conditions.

First, to identify the brain regions related to rhythm processing, we contrasted the brain activity during the RHY tasks with that during the corresponding NUM tasks. The results for the encoding and retrieval phases for each effector are summarized in Fig 3 and Table A in S1 File. During rhythm encoding, we found brain activations in the bilateral inferior frontal gyri (IFGs), right inferior parietal lobules (IPL) and left cerebellum (CB) during rhythm encoding in the right finger, left finger and right foot conditions. The right IPL was also activated by the mouth. The left IPL was activated by the right and left finger. Almost the same brain regions were activated during rhythm retrieval. In addition to these brain regions, the bilateral supplementary motor areas (SMA) were activated by the right finger, left finger and right foot.

**Fig 4:**
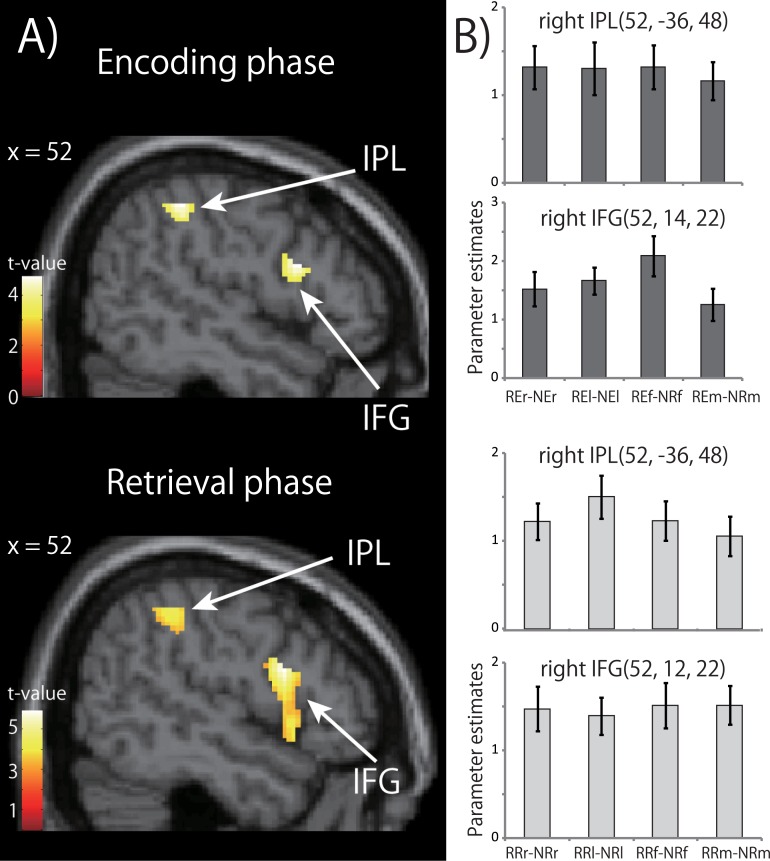
A) Results of conjunction analysis. Cortical brain regions in common activated by all four effectors showed significant activations in the rhythm working memory tasks compared to the number working memory tasks (threshold: *P* < 0.05; FWE-corrected of the clusters). B) Response profile for each activation peak. Response profiles are shown for the right IPL and IFG in the encoding and retrieval phases. From these peaks, parameter estimates were extracted for all (rhythm—number) contrasts. Error bars indicate the standard error of the mean. Abbreviations: IFG, inferior frontal gyrus; IPL, inferior parietal lobule. REr represents Rhythm-Encoding-right finger condition. See Methods for details of the contrasts.

Next, to elucidate brain regions related to rhythm processing independently of the effector used, we performed a conjunction analysis across all four effector conditions, using a contrast (RXr—NXr) ∩ (RXl—NXl) ∩ (RXf—NXf) ∩ (RXm—NXm) in each phase, where X is the E or R phase. The results are shown in Fig 4 and Table 1. The right IFG and IPL were in common activated by all four effectors, and exhibited significant activations in the RHY tasks compared to the NUM tasks during both encoding and retrieval phases (Fig 4A). The activation in the right IFG during the encoding phase met the criterion only at the peak level (*P* < 0.05, corrected), but we regarded the activation as significant based on the *a priori* hypothesis that the right IFG was involved in rhythm encoding and that the peak coordinate of the cluster corresponded to that of a previous experiment [9].

**Fig 5:**
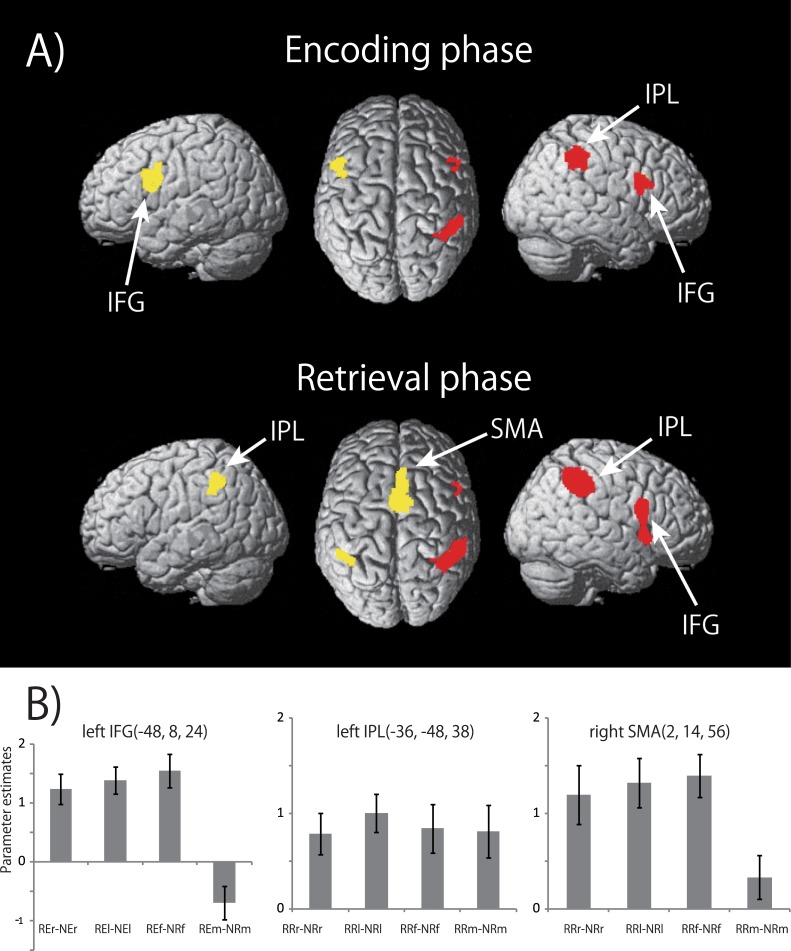
A) Results of second conjunction analysis. Cortical brain regions demonstrating common brain activations by the right index finger, the left index finger and the right foot (except for the mouth), showed significant activations in the rhythm working memory tasks compared to the number working memory tasks (threshold: *P* < 0.05; FWE-corrected of the clusters). Red areas are consisted with the cortical regions, which were in common activated by all four effectors (Fig 4A). Yellow areas represent the additional regions in common activated by the three effectors, resulting from this second conjunction analysis. B) Response profiles are shown for the left IFG during rhythm encoding and the left IPL and the SMA during rhythm retrieval. The peak voxels of response profiles were obtained from the results of mean activations across all effectors using a contrast [(RXr—NXr) + (RXl—NXl) + (RXf—NXf) + (RXm—NXm)]. See Fig 4 legend for details of the response profile.

As shown in Fig 3, there are several differences between brain activations in the mouth condition and the other effector conditions. For example, the bilateral IFGs and the left CB were activated by the right finger, the left finger, and the right foot, whereas these regions were not activated by the mouth during rhythm encoding. Furthermore, neither was the SMA activated by the mouth during rhythm retrieval. On the other hand, the bilateral STGs were activated only by the mouth during rhythm retrieval. Similar cortical regions were activated by the right finger, left finger and right foot. Next, to survey common activated brain regions by the right finger, left finger and right foot, we conducted an additional conjunction analysis using the contrasts [(RXr—NXr) ∩ (RXl—NXl) ∩ (RXf—NXf)]. The results are summarized in Fig 5 and Table 1. The left IFG showed a significant activation during rhythm encoding whereas the left IPL and the right SMA somewhat extending to the left showed significant activation during rhythm retrieval by the right finger, left finger and right foot, but not by the mouth (Fig 5A).

In addition, the right precuneus was activated by the left finger, the right foot, and the mouth, but not by the right finger during rhythm encoding. The left cerebellum was activated by the right and left finger, but not by the right foot and the mouth.

To reveal the response characteristics of the effector-independent and effector-dependent regions, we calculated parameter estimates for each phase during the tasks. The results are shown in Figs 4B and 5B. The locations that showed peak activations in the right IFG and right IPL were obtained from the result of the conjunction analysis of [(RXr—NXr) ∩ (RXl—NXl) ∩ (RXf—NXf) ∩ (RXm—NXm)]. The peak locations in the left IFG, left IPL and right SMA were obtained from the result of the mean activation for all effectors using a contrast [(RXr—NXr) + (RXl—NXl) + (RXf—NXf) + (RXm—NXm)]. The effects of the EFFECTOR (right finger, left finger, right foot or mouth) were examined by a one-way ANOVA. No significant main effect was observed in the response profiles in the right IFG and right IPL (effector-independent regions) and left IPL (effector-dependent region). The effect of EFFECTOR was significant in the left IFG and the SMA (effector-dependent region) (F_(3,88)_ = 15.86, *P* < 0.0001 and F_(3,88)_ = 3.671, *P* = 0.015, respectively). A post-hoc analysis of multiple comparisons revealed that, in the left IFG, the parameter estimates for the mouth were significantly lower than those for the other effectors, and lower than those estimated for the left finger and right foot in the SMA.

In summary, the right IFG and IPL showed effector-independent brain activations during both rhythm encoding and retrieval, whereas the left IFG showed effector-dependent activation during rhythm encoding, and the left IPL and the SMA showed the effector-dependent activations during rhythm retrieval.

#### Effector-specific activation during rhythm retrieval

To identify the brain regions that were specific to the motor effector, we contrasted the rhythm-related activity in each effector condition with those in the other conditions. We found left finger-specific activation in the right postcentral gyrus (somatosensory cortex), foot-specific activation in the left postcentral gyrus, and mouth-specific activations in the bilateral pre/postcentral gyri (primary motor and somatosensory cortices), superior temporal gyrus (STG), superior frontal gyrus and right hippocampus (Figure A and Table B in S1 File). We found no significant right finger-specific activations. In contrast to rhythm retrieval, no significant effector-specific and rhythm-related brain activations were observed during rhythm encoding.

#### Functional connectivity during rhythm encoding

**Fig 6:**
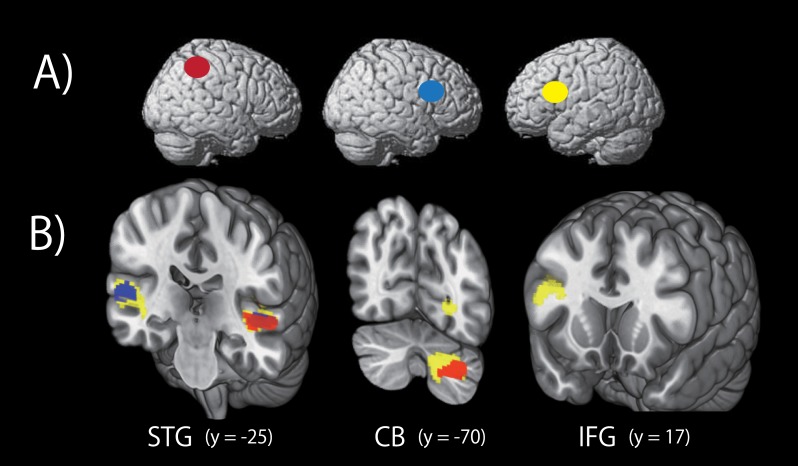
Functional connectivity during rhythm encoding. A) Three seed regions; right IPL (red), right IFG (blue), and left IFG (yellow) from left to right. B) Brain areas exhibiting significantly increased coupling with the seed regions in the presence of rhythm encoding. Colored areas show significant coupling with the seed region in the same color. Red, blue, and yellow regions show significant functional connectivity with the right IPL, right IFG, and let IFG, respectively. Threshold: *P* < 0.05; FWE-corrected of the clusters. Abbreviations: CB, cerebellum; IFG, inferior frontal gyrus; STG, superior temporal gyrus.

To investigate functional connectivity during rhythm encoding, we conducted PPI analyses. The right IFG and IPL were used as the seed regions for the effector-independent regions, and the left IFG was used as the seed region for the effector-dependent regions. These results are summarized in Fig 6 and Table 2. The seed regions were defined in each participant, based on the effector-independent or effector-dependent brain activations resulting from two conjunction analyses (Table 1). For the effector-independent regions, we found a significant increase in connectivity between the right IFG and bilateral STG under the modulation of the RHY tasks compared to the NUM tasks (Fig 6B, blue areas). The right IPL seed showed a significant connectivity with the left STG and cerebellum (Fig 6B, red areas). For the effector-dependent region, the left IFG seed showed significant connectivity with the bilateral STG, right IFG and left cerebellum (Fig 6B, yellow areas).

**Table 2:** Functional connectivity analysis.

effector	Brain region	size	*p*	*T*	xyz (MNI)
**Encoding phase**					
Seed region: Rt. IFG (52 14 22)					
all	Rt. superior temporal gyrus	540	< 0.001	4.83	56–26 4
	Lt. superior temporal gyrus	641	< 0.001	4.68	-42–38 10
Seed region: Rt. IPL (52–36 48)					
all	Lt. cerebellum (VI)	366	0.003	5.21	-30–68–40
	Lt. superior temporal gyrus	630	< 0.001	4.74	-58–20 0
Seed region: Lt. IFG (-50 8 22)					
all	Lt. cerebellum (VI)	832	< 0.001	7.83	-16–74–36
	Rt. superior temporal gyrus	1182	< 0.001	6.23	56–26 4
	Rt. inferior frontal gyrus	364	0.002	5.62	36 8 28
	Lt. superior temporal gyrus	1605	< 0.001	5.44	-38–34 12
**Retrieval phase**					
Seed region: Rt. IPL (52–36 48)					
Lt. finger	Rt. postcentral gyurs	604	< 0.001	5.73	56–26 42
	Lt. cerebellum (V)	519	< 0.001	5.54	-20–58–24
foot	Rt. precentral gyrus	2633	< 0.001	6.31	14–14 46
	Rt. postcentral gyrus			5.98	20–38 66
	Rt. supplementary motor area			5.97	6–20 54
	Lt. supplementary motor area			5.49	-4–2 44
	Lt. superior parietal lobule			4.98	-14–42 68
	Lt. postcentral gyrus			4.96	-10–40 68
	Lt. parietal opeculum	198	198	4.89	-48–30 22
Seed region: Lt. IPL (-48–44 46)					
Rt. finger	Rt. cerebellum (V)	493	< 0.001	5.29	4–64–24
Lt. finger	Rt. caudate	1358	< 0.001	6.97	18 16 6
	Rt. thalamus				16–12 6
	Lt. cerebellum (VI)	695	< 0.001	6.90	-30–58–34
	Lt. thalamus	521	< 0.001	5.04	-12–16 8
	Rt. supplementary motor area	407	< 0.001	4.98	0–6 60
	Lt. supplementary motor area				-4–12 64
	Rt. postcentral gyurs	304	0.003	4.81	36–28 52
	Rt. precentral gyrus			4.16	42–20 50
foot	Rt. postcentral gyrus	182	0.019	5.21	26–30 64
	Rt. precentral gyrus			4.34	18–26 58
Seed region: Rt. SMA (2 14 56)					
Lt. finger	Lt. precentral gyrus	484	0.001	5.19	-26–12 48
	Rt. postcentral gyrus	661	< 0.001	4.83	34–28 52
	Rt. inferior parietal lobule			4.68	42–38 46
	Rt. precentral gyrus			4.27	20–24 62
	Lt. cerebellum (V)	216	0.029	4.51	-14–56–22
foot	Rt. superior parietal lobule/ post central gyrus	345	0.001	6.21	16–48 66
	Rt. precentral gyrus			4.59	16–28 66
	Lt. superior parietal lobule/ post central gyrus	185	0.020	5.75	-16–34 58
	Rt. supplementary motor area	247	0.005	4.48	2–14 58

#### Functional connectivity during rhythm retrieval

**Fig 7:**
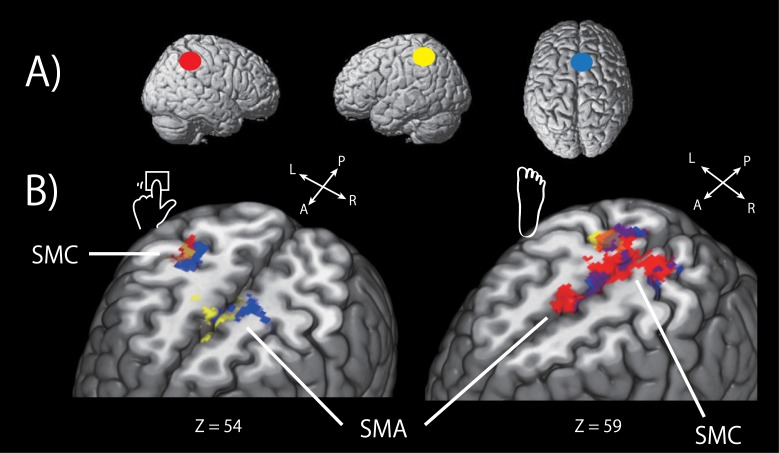
Functional connectivity during rhythm retrieval. A) Three seed regions; right IPL (red), left IPL (yellow), SMA (blue) from left to right. B) The brain areas exhibiting significantly increased coupling with the seed regions in the presence of rhythm retrieval. Red, yellow, and blue regions show significant functional connectivity with the right IPL, left IPL, and SMA, respectively. Right panel: left finger condition, left panel: foot condition. Threshold: *P* < 0.05; FWE-corrected of the clusters. Abbreviations: SMA, supplementary motor area; SMC, somatosensory/motor cortex.

We also investigated functional connectivity during rhythm retrieval using PPI analyses. The results are summarized in Fig 7 and Table 2. In brief, for the effector-independent regions, there were no significant changes in connectivity between the right IFG and other regions during rhythm retrieval. The right IPL showed significant increases in functional connectivity with the right pre/postcentral gyri and left cerebellum caused by the left finger (Fig 7B, red areas in the left panel), and with the bilateral pre/postcentral gyri, SMA, and left parietal operculum caused by the right foot (Fig 7B, red areas in the right panel). For the effector-dependent region, the left IPL showed significant increases in functional connectivity with the SMA, basal ganglia, right pre/postcentral gyri, and left cerebellum triggered by the left finger (Fig 7B, yellow areas in the left panel), and with the right postcentral gyrus evoked by the right foot (Fig 7B, yellow areas in the right panel). The PPI analysis of the SMA seed revealed increases in connectivity with the left precentral gyrus, cerebellum and right pre/postcentral gyrus induced by the left finger (Fig 7B, blue areas in the left panel), and with the bilateral pre/postcentral gyri and superior parietal lobules caused by the right foot (Fig 7B, blue areas in the right panel).

## Discussion

The aim of the present study was to elucidate neural correlates of the temporal and motor representation of rhythm. The behavioral analysis revealed that the accuracy of the rhythm tasks was indistinguishable among the four effectors. This result is consistent with the viewpoint that the accuracy of performance was not specific to the effector at the early stages of motor learning [11] and in rhythm reproduction [6], suggesting a common mechanism of rhythm processing across the effectors.

We demonstrated that the right IFG and the IPL were activated during both rhythm encoding and retrieval, regardless of the utilized effectors. On the other hand, the left IFG exhibited a significant activation during rhythm encoding, and the left IPL and SMA showed significant activations during rhythm retrieval. The neural representation of motor sequences should depend on effectors, while that of temporal sequences would be effector-independent. Based on this assumption, we discuss below the functions of activated regions.

Although the basal ganglia and cerebellum also have been thought to be cardinal regions in the rhythm processing [2, 4, 5, 6, 9, 12, 13, 14, 15], the functions of these brain regions are not discussed here.

### 4.1 Rhythm encoding

The right IFG was activated during rhythm encoding, regardless of the employed effectors, and the left IFG was activated during rhythm encoding in the right finger, left finger, and right foot conditions (but not in the mouth condition). Several brain imaging studies have reported rhythm-related activations in the right, left or bilateral IFGs [12, 16, 17, 18]. These reports have suggested that the IFGs are involved in rhythm processing. The IFG activation observed here was restricted to the opercular region (BA 44). This region is implicated in syntax processing of the language and music [19, 20, 21], and in memory of structured sequences [22]. These results suggest that during rhythm encoding the IFG regions play a crucial role in organizing perceived sound elements into a structured temporal sequence of rhythm.

Why did the left IFG show no significant activations in the mouth condition? The low rhythm-related activity of the left IFG in the mouth condition (Fig 5) caused by extremely high activation during encoding phase of the NUM task rather than that of the RHY task. The IFG is closely related to mouth movement, including internal speech [23]and motor rehearsal of articulation [24]. Consequently, the left IFG exhibited no significant activations during rhythm encoding only in the mouth condition (Fig 3). Thus, the left IFG as well as the right IFG probably play an effector-independent role in rhythm processing.

Although few previous studies have reported IPL activations related to rhythm perception or reproduction [9], some brain imaging studies have suggested the involvement of the bilateral IPL regions in time perception [25, 26], and especially that of the right IPL in temporal prediction and production [27]. In addition, dysfunction of the right inferior parietal cortex can induce impairment in temporal information processing [28, 29, 30, 31]. Furthermore, Grahn and Rowe (2013) have reported the activation in the right inferior parietal cortex during the detection of a musical beat in irregular rhythms, which have no regularity and require the encoding of each interval individually, compared to that of regular rhythms. These results suggest that the right IPL is involved in representing temporal information of rhythm, especially in the perceptual time of each interval of rhythm sequences.

Importantly, these areas showed the rhythm-related activations independent of the motor effectors, but also independent of sensory modalities: auditory or visual [9]. Thus, in conclusion, the bilateral IFG regions and right IPL may play an important role in the representation of temporal sequences during rhythm encoding.

### 4.2 Rhythm retrieval

The right IFG and right IPL exhibited effector-independent activations during rhythm retrieval. When a rhythm was reproduced, the temporal representation of the rhythm in the right IFG and right IPL was probably employed. The bilateral inferior parietal cortices are typically activated during finger sequence learning [32, 33, 34], and may play a role in the sensory-motor transformation of temporal and spatial relation [35]. We could easily image the movement of a finger or a foot, whereas imaging of the mouth is more difficult, because we could not observe the actual movements of our lips. In addition, the movement of a finger or a foot is a spatial task, unlike the movement of the mouth. The difficulty in transformation of temporal representation into a motor (spatial) representation could affect the activation in the left IPL among effectors.

Activations in the SMA have been reported frequently in relation to rhythm processing [2, 3, 5, 7, 10, 12, 13,15]. Notably, Bengtsson and colleague (2005) have reported the effector-independent activation in the SMA during rhythm reproduction, which was consistent with our present result. Interestingly, patients with SMA lesions showed impairment in rhythm reproduction from memory [36], suggesting that the SMA is specifically involved in the retrieval of rhythm. In addition, neurons in the monkey SMA encode motor sequences [37, 38]. Thus, the SMA is implicated in representing motor sequences during rhythm retrieval. The SMA has been thought to be involved in the motor imagery or rehearsal of sequential movements [34, 39]. The difficulty in imaging movements on spatial maps among effectors may be the reason why the effector-dependent regions show the lowest activations under the mouth condition.

We found rhythm-related and effector-specific activations in the contralateral somatosensory cortices corresponding to the left finger and the right foot. This somatotopical localization may reflect involvement in the sensory feedback of rhythmic motor control [40]. We found no significant right finger-specific activations, suggesting a lower load of the RHY task for the right finger compared to the other effectors. The mouth-specific activations were observed in the bilateral somatosensory/motor cortices and STGs. The STG has been implicated in auditory processing, including rhythm [41, 42]. For the mouth, the participants could perceive their own voice beyond the headphone, and judge if the rhythmic pattern matched to the memorized rhythm. Thus, the activations in the bilateral STG regions were probably related to the sensory monitoring of their rhythmic vocalization. The somatosensory/motor cortices also may reflect motor control and sensory feedback of the mouth movements.

### 4.3 Neural networks involved in representation of temporal and motor sequence

**Fig 8:**
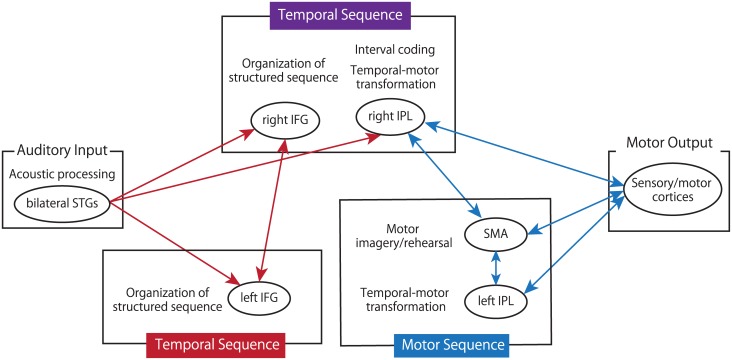
Hypothetical model of working memory of rhythm. During rhythm encoding, the bilateral inferior frontal gyri (IFGs) and right inferior parietal lobule (IPL) receive auditory rhythm information from the superior temporal gyri (STGs), and encode the information as a temporal structured sequence. The temporal sequence of the rhythm is stored in the right fronto-parietal network. During rhythm retrieval, the IPLs and supplemental motor area (SMA) transform the temporal sequence into the motor sequence of the rhythm, and reproduce the rhythm through various effectors while monitoring the output with the feedback signals from the somatosensory/motor cortices. Red lines indicate functional connectivities during rhythm encoding, and the blue lines indicate those during rhythm retrieval. The filled rectangles indicate the estimated functional units of working memory of rhythm; encoding (red), retrieval (blue) and both (purple).

The PPI analysis revealed that the bilateral IFG regions and right IPL showed significant increases in connectivity with the bilateral STGs during rhythm encoding. The anatomical projection from the superior temporal regions to the inferior frontal regions was revealed in monkeys [43, 44] and humans [45, 46]. Moreover, anatomical connections between the IPL and the posterior STG have also been reported in humans [46]. In addition, we found a functional connectivity between the right IFG and left IFG. These results suggest that the bilateral IFGs and right IPL receive acoustic information about each element of the auditory sequence from the STGs, and then the bilateral IFGs transform the information into temporal structures of rhythm by interacting with each other during rhythm encoding (Fig 8).

During rhythm retrieval, the bilateral IPLs and SMA exhibited significant increases in connectivity with each other and with the somatosensory/motor cortices. The parietal cortex has been implicated in the integration between the sensory feedback and the efference copy [47]. For precise reproduction of rhythm, monitoring of the performance from the somatosensory feedback signal, and online movement correlation should be necessary. These data suggest that the bilateral IPLs and SMA receive information about the temporal sequences of rhythm from the right IFG and right IPL, and transform it into motor sequences of rhythm (Fig 8). Then, the bilateral IPLs and SMA reproduce the rhythm sequences by interacting with the somatosensory/motor cortices (Fig 8).

## Conclusion

In the present study, we explored the neural networks associated with representing the temporal and motor sequences of rhythm. Here, we propose a new model of rhythm processing as follows. During rhythm encoding, the bilateral IFGs and right IPL receive auditory rhythm information from the STGs, and encode the information as temporal structured sequences. Then, the temporal sequence of rhythm is stored in the right fronto-parietal network. During rhythm retrieval, the IPLs and SMA transform the temporal sequence into the motor sequence of the rhythm, and reproduce the rhythm through various effectors while monitoring the output with the feedback signals from the somatosensory/motor cortices. Our model provides a novel view about the entire processes of rhythm reproduction and may be applied to neural mechanisms underlying long-term acquisition of rhythm performance.

## Supporting Information

S1 File **Figure A**. Cortical brain regions showing effector-specific, rhythm-related activations during rhythm retrieval. **Table A**. Increased activations in the RHY task compared to the NUM task in each effector condition. **Table B**. Effector-specific and rhythm-related activations during rhythm retrieval. **Text A**. Activations correlated with behavioral performance.
(DOC)
