# Supplementary material for: Temporal and Motor Representation of Rhythm in Fronto-Parietal Cortical Areas: An fMRI Study
Source: PLoS One. 2015 Jun 15;10(6):e0130120. doi: 10.1371/journal.pone.0130120 (PMC4468110; doi:10.1371/journal.pone.0130120)
Supplement: S1 File — Figure A. Cortical brain regions showing effector-specific, rhythm-related activations during rhythm retrieval. Table A. Increased activations in the RHY task compared to the NUM task in each effector condition. Table B. Effector-specific and rhythm-related activations during rhythm retrieval. Text A. Activations correlated with behavioral performance. (DOC) [file pone.0130120.s001.doc]

**Figure A. Cortical brain regions showing effector-specific, rhythm-related activations during rhythm retrieval.**

A) left finger, B) foot, and C) mouth conditions. No significant effector-specific activations were induced by the right finger. Abbreviations: SC: somatosensory cortex; SMC, somatosensory/motor cortex; STG, superior temporal gyrus. Threshold: *P* < 0.05, FWE-corrected.


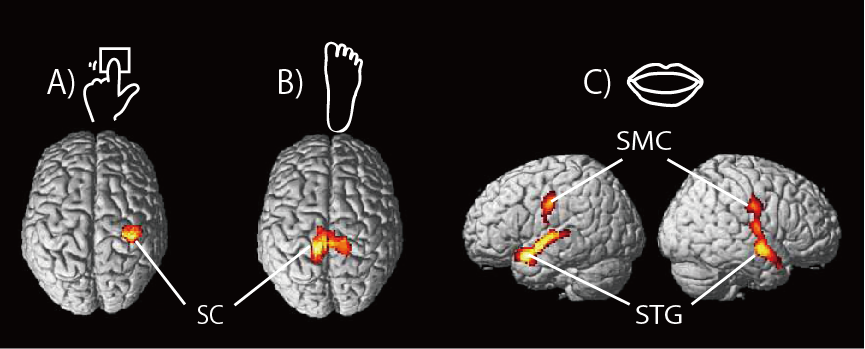


**Table A.** Increased activations in the RHY task compared to the NUM task in each effector condition.

| phase | effector | Brain region | size | *p* | *T* | xyz (MNI) |
| --- | --- | --- | --- | --- | --- | --- |
| E | right finger | Rt. inferior parietal lobule | 700 | < 0.001 | 6.73 | 46 -42 48 |
|  |  | Rt. inferior frontal gyrus | 307 | 0.010 | 5. 73 | 50 10 24 |
|  |  | Lt. caudate | 439 | 0.002 | 5.57 | -18 10 10 |
|  |  | Lt. inferior parietal lobule | 411 | 0.002 | 5.40 | -40 -48 46 |
|  |  | Lt. inferior frontal gyrus | 361 | 0.005 | 5.33 | -50 6 22 |
|  |  | Lt. cerebellum (VI) | 229 | 0.031 | 5.05 | -28 -66 -32 |
|  | left finger | Lt. inferior frontal gyrus | 465 | 0.001 | 8.99 | -46 6 20 |
|  |  | Rt. inferior frontal gyrus | 534 | 0.001 | 7.32 | 52 12 22 |
|  |  | Rt. inferior parietal lobule | 911 | < 0.001 | 5.81 | 40 -46 42 |
|  |  | Rt. precuneus |  |  | 5.49 | 22 -66 42 |
|  |  | Lt. cerebellum (VI) | 242 | 0.026 | 5.53 | -26 -66 -34 |
|  |  | Lt. inferior parietal lobule | 224 | 0.034 | 4.95 | -36 -48 40 |
|  | foot | Rt. precuneus | 387 | 0.003 | 6.88 | 22 -72 50 |
|  |  | Rt. inferior frontal gyrus | 279 | 0.012 | 6.41 | 48 14 28 |
|  |  | Lt. cerebellum (VI) | 620 | < 0.001 | 5.85 | -20 -72 -30 |
|  |  | Lt. inferior frontal gyrus | 476 | 0.001 | 5.76 | -48 10 24 |
|  |  | Rt. inferior parietal lobule | 287 | 0.011 | 5.48 | 50 -38 48 |
|  | mouth | Rt. inferior parietal lobule | 248 | 0.013 | 5.38 | 52 -36 48 |
|  |  | Rt. precuneus | 188 | 0.037 | 5.08 | 26 -64 34 |
| R | right finger | Rt. inferior parietal lobule | 846 | < 0.001 | 6.91 | 48 -38 48 |
|  |  | Rt. inferior frontal gyrus | 544 | 0.001 | 5.78 | 52 12 22 |
|  |  | Lt. supplementary motor area | 528 | 0.001 | 5.30 | -4 0 58 |
|  |  | Rt. supplementary motor area |  |  | 4.96 | 8 24 50 |
|  |  | Rt. cerebellum (VI) | 806 | < 0.001 | 4.93 | 32 -58 -34 |
|  |  | Lt. inferior parietal lobule | 431 | < 0.001 | 4.88 | -48 -42 48 |
|  | left finger | Rt. inferior parietal lobule | 1922 | < 0.001 | 9.65 | 50 -44 48 |
|  |  | Lt. cerebellum (VI) | 1557 | < 0.001 | 7.60 | -30 -60 -34 |
|  |  | Rt. thalamus | 893 | < 0.001 | 7.19 | 14 -2 6 |
|  |  | Lt. superior temporal gyrus | 691 | < 0.001 | 7.01 | -48 12 -4 |
|  |  | Rt. inferior frontal gyrus | 896 | < 0.001 | 6.85 | 50 12 16 |
|  |  | Lt. supplementary motor area | 1537 | < 0.001 | 6.35 | -6 -8 64 |
|  |  | Rt. supplementary motor area |  |  | 5.45 | 4 0 66 |
|  |  | Lt. inferior parietal lobule | 696 | < 0.001 | 5.81 | -40 -50 42 |
|  |  | Lt. caudate | 532 | < 0.001 | 5.55 | -22 -12 20 |
|  | foot | Rt. inferior frontal gyrus | 1367 | < 0.001 | 7.24 | 48 30 6 |
|  |  | Rt. supplementary motor area | 2169 | < 0.001 | 7.11 | 4 0 62 |
|  |  | Lt. supplementary motor area |  |  | 6.34 | -4 -18 64 |
|  |  | Lt. inferior frontal gyrus | 502 | 0.001 | 6.36 | -42 24 4 |
|  |  | Rt. inferior parietal lobule | 743 | < 0.001 | 5.91 | 50 -46 48 |
|  |  | Rt. cerebellum (VI) | 417 | 0.003 | 5.69 | 2 -50 -14 |
|  | mouth | Lt. superior temporal gyrus | 1815 | < 0.001 | 8.09 | -46 12 -12 |
|  |  | Lt. inferior frontal gyrus |  |  | 6.29 | -54 8 30 |
|  |  | Rt. inferior frontal gyrus | 740 | < 0.001 | 8.04 | 52 8 20 |
|  |  | Rt. superior temporal gyrus |  |  | 5.11 | 52 0 -10 |
|  |  | Rt. inferior parietal lobule | 218 | 0.046 | 4.97 | 54 -36 46 |

**Table B. Effector-specific and** rhythm-related activations during rhythm retrieval

| Effector | Brain region | size | *p* | *T* | xyz (MNI) |
| --- | --- | --- | --- | --- | --- |
| left finger | Rt. postcentral gyrus | 436 | 0.006 | 5.57 | 38 -28 52 |
| Foot | Lt. postcentral gyrus | 916 | < 0.001 | 5.82 | -10 -36 66 |
|  | Rt. postcentral gyrus |  |  | 4.42 | 14 -38 66 |
| mouth | Lt. superior temporal gyrus | 1876 | < 0.001 | 5.33 | -54 -4 -8 |
|  | Lt. superior frontal gyrus | 657 | 0.001 | 5.26 | -8 58 22 |
|  | Rt. superior frontal gyrus |  |  | 4.31 | 6 58 22 |
|  | Rt. superior temporal gyrus | 968 | < 0.001 | 5.17 | 56 2 -14 |
|  | Rt. precentral gyrus |  |  | 4.47 | 54 -10 34 |
|  | Rt. hippocampus | 288 | 0.031 | 4.85 | 30 -10 -20 |
|  | Lt. precentral gyrus | 387 | 0.010 | 4.32 | -54 -12 36 |

**Text A. Activations correlated with behavioral performance**

Methods:

To assess the changes in neural activity directory related to the accuracy of rhythm reproduction in the RHY tasks, we examined the relationship between the GIR index values and parameter estimates for all participants, at each peak location of the effector-independent and dependent activations obtained from the conjunction analyses. We also conducted a parametric analysis using SPM8 to examine the relationship between BOLD signal and GIR index value of each RHY trial as a parameter modulator.

Results:

We performed a regression analysis for all peak locations of effector-independent and dependent activations (Table 1) using the GIR index as a regressor. We found no significant correlation between brain activity and the GIR index value (Pearson' s correlation coefficient, *P* > 0.05) during both encoding and retrieval. We further investigated the relationship between the BOLD signal and the GIR index value for each trial of the RHY tasks as a parameter modulator. However, no brain regions exhibited a significant relationship between the BOLD signal and the GIR index value.
